# Neurogranin stimulates Ca^2+^/calmodulin-dependent kinase II by suppressing calcineurin activity at specific calcium spike frequencies

**DOI:** 10.1371/journal.pcbi.1006991

**Published:** 2020-02-12

**Authors:** Lu Li, Massimo Lai, Stephen Cole, Nicolas Le Novère, Stuart J. Edelstein

**Affiliations:** 1 Babraham Institute, Cambridge, United Kingdom; 2 Quantitative Systems Pharmacology, CERTARA, Canterbury, United Kingdom; 3 Cambridge Systems Biology Centre, University of Cambridge, Cambridge, United Kingdom; 4 aSciStance Ltd, Cambridge, United Kingdom; 5 Scipio bioscience, Paris, France; Instytut Biologii Doswiadczalnej im M Nenckiego Polskiej Akademii Nauk, POLAND

## Abstract

Calmodulin sits at the center of molecular mechanisms underlying learning and memory. Its complex and sometimes opposite influences, mediated via the binding to various proteins, are yet to be fully understood. Calcium/calmodulin-dependent protein kinase II (CaMKII) and calcineurin (CaN) both bind open calmodulin, favoring Long-Term Potentiation (LTP) or Depression (LTD) respectively. Neurogranin binds to the closed conformation of calmodulin and its impact on synaptic plasticity is less clear. We set up a mechanistic computational model based on allosteric principles to simulate calmodulin state transitions and its interactions with calcium ions and the three binding partners mentioned above. We simulated calcium spikes at various frequencies and show that neurogranin regulates synaptic plasticity along three modalities. At low spike frequencies, neurogranin inhibits the onset of LTD by limiting CaN activation. At intermediate frequencies, neurogranin facilitates LTD, but limits LTP by precluding binding of CaMKII with calmodulin. Finally, at high spike frequencies, neurogranin promotes LTP by enhancing CaMKII autophosphorylation. While neurogranin might act as a calmodulin buffer, it does not significantly preclude the calmodulin opening by calcium. On the contrary, neurogranin synchronizes the opening of calmodulin’s two lobes and promotes their activation at specific frequencies. Neurogranin suppresses basal CaN activity, thus increasing the chance of CaMKII trans-autophosphorylation at high-frequency calcium spikes. Taken together, our study reveals dynamic regulatory roles played by neurogranin on synaptic plasticity, which provide mechanistic explanations for opposing experimental findings.

## Author summary

How our brains learn and remember things lies in the subtle changes of the strength of the connections between brain cells, the so-called synaptic plasticity. At the level of the recipient neuron, some of the information is encoded into patterns of intracellular calcium spikes. Calmodulin, a small bi-lobed protein whose conformation is regulated by the binding of calcium ions, decodes these signals and modulates the activity of specific binding partners. Two key regulators, calcineurin and calcium/calmodulin-dependent protein kinase II, which respectively weakens or strengthens synaptic connections, bind both lobes of calmodulin in its open form, stabilized by calcium. On the contrary, neurogranin binds preferentially to one lobe of calmodulin when in the closed form, destabilized by calcium. It was thus initially suggested that neurogranin would inhibit calmodulin activation and decrease synaptic plasticity. However, past research showed that neurogranin sometimes actually enhances synaptic plasticity, though the mechanism was unclear. Our computational models showed that neurogranin synchronizes the activation of the two lobes of calmodulin, favoring opening at high-frequency calcium spikes. By doing so, neurogranin increases the impact of calmodulin on calcium/calmodulin-dependent protein kinase II and reduces its effect on calcineurin, resulting in a strengthening of synaptic connections.

## Introduction

Calmodulin (CaM) is a small ubiquitous calcium-binding protein whose cellular activity is mediated via the binding of a large array of target proteins. CaM comprises two lobes, each binding two calcium ions, that can undergo transitions between a closed (T) and an open (R) conformation. Calcium binding shifts the transition towards the open form [1, 2]. Some CaM-binding proteins favor the open conformation while others tend to bind to the closed conformation [3–8].

In neurons, CaM plays a crucial role in mediating calcium regulation of synaptic plasticity, a mechanism underlying learning and memory. Among its main binding partners, Ca^2+^/calmodulin-dependent protein kinase II (CaMKII) and calcineurin (CaN) preferably bind to the open (R) form of CaM, and compete to facilitate long-term potentiation(LTP) or long-term depression (LTD) respectively [2, 9, 10]. On the contrary, neurogranin (Ng), an IQ domain-containing protein, binds preferentially to the closed (T) state, therefore sequestering CaM from its other targets in the absence of calcium [11–13]. Ng’s role in the regulation of synaptic plasticity is still under debate. Ng seems to facilitate LTP induction at high frequency electrical pulses [14–18]. However, it has also been reported to produce the opposite effect [19]. Deciphering the mechanisms underlying Ng’s opposing functions, and how Ng coordinates the activities of CaMKII and CaN in response to calcium signals, are crucial for understanding how CaM regulates brain function and dysfunction.

Although structurally similar, the two CaM lobes display different properties. The carboxy-terminal (C) lobe possesses higher calcium binding affinities but slow kinetics while the amino-terminal (N) lobe has lower calcium binding affinities but faster binding kinetics [6, 20–23]. In addition, the lobes exhibit a significant degree of structural autonomy [24, 25]. CaM lobes undergo constant transitions between the open and closed conformations, transitions regulated by the binding of both calcium ions and target proteins. The key to understanding CaM function lies in the mechanisms underlying the differential activation of its targets and how binding these targets feeds back to its conformational changes.

The two lobes contribute unevenly to target binding. Targets binding preferentially to the closed state, especially Ng, interact predominantly with the C lobe [5, 7, 13, 26–28]. Whereas targets binding preferentially to the open state, such as CaMKII and CaN, contact both domains [4, 6, 8, 29, 30]. Accordingly, binding these targets affects differentially the conformational shifts of both lobes, as indicated by the modification of apparent calcium binding affinities [5, 6, 21, 26]. Targets stabilize the conformation for which they have the highest affinity (i.e. the complexes have the lowest free energy). Reciprocal influences between target binding and calcium binding often exhibit lobe specificity as well [5, 31–33]. Therefore, how CaM regulates the function of its binding partners, including CaMKII, CaN, and Ng, is tightly linked with its structure and the functional differences between its lobes.

A number of computational models have been developed to study the regulation of bidirectional synaptic plasticity [34–39]. While these studies have advanced our understanding of how CaMKII responds to different shapes of calcium signals, they modeled the activation of CaM either using phenomenological descriptions such as the Hill or the Adair-Klotz equations or using sequential bindings of calcium ions. Kubota *et al*. took one step further by considering CaM’s lobe differences. They modeled the cooperativity between calcium-binding sites on each lobe as consecutive bindings with increased affinities [32]. We previously proposed a mechanistic description of CaM within an allosteric framework [2, 10], but which did not take the role of Ng into account. A few models have advanced our understanding on how Ng facilitates LTP, putting forward Ng’s dynamic regulation of CaM concentration in the post-synaptic density (PSD) and the reshaping of free calcium spikes as proposed mechanisms [40–42]. Using mathematical modeling, Romano *et al*. [43] recently showed that Ng facilitates CaMKII activation at a 100 Hz-tetanus stimulation. However, they failed to show why, at the same time, Ng hinders LTP induction, as observed by the same experimental group [19].

In this paper, we present a detailed mechanistic model of CaM in the context of synaptic plasticity and its interactions with CaMKII, CaN, and Ng, based on the allosteric framework and our previous hemiconcerted CaM model [25]. The four calcium-binding sites were explicitly modeled and each lobe of CaM undergoes independent state transitions. CaM binding to its targets depends on the conformation of CaM rather than the number of calcium ions bound to it, as the status of calcium saturation does not correlate with a specific conformation [44–47]. In addition, we systematically re-estimated the allosteric parameters concerning the two CaM lobes together, as well as the kinetic constants, based on steady states and calcium chelating time course experimental data of full-length CaM. We simulated a wide range of calcium-spike frequencies in a wild type context and for a Ng knock-out situation.

We show differences between CaM’s lobes in response to calcium signals. We show how they contribute to, but also are influenced by, the differential binding of CaM’s targets. We show that Ng is not merely a CaM buffer protein, but it can adjust the activation of CaM at spike frequencies that maximize CaMKII activation. Ng synchronizes the opening of the two CaM lobes. Ng’s regulation of LTP induction depends on the concentration of CaN, and its quantity in relation to CaM.

## Materials and methods

### Model structure

We built a mechanistic mathematical model to describe the conformational changes of CaM lobes, calcium bindings, and CaM interaction with binding partners. We expanded and re-parameterized our previous hemiconcerted allosteric model of CaM by Lai *et al*. [25]. The major differences between our model and other published CaM models are: 1) We do not assume sequential bindings of calcium ions to the four binding sites. Instead, each calcium-binding site on CaM is independent and independent from the binding of protein partners.2) A target binds to CaM that has a specific conformation, other than a specific number of calcium ions bound to it. Similarly, a target binds to CaM that has the preferred conformation, with the same affinity regardless of the number of calcium ions bound. 3) We do not modify intrinsic affinities for CaM binding calcium to mimic the apparent affinity changes exerted by CaM-binding targets. Calcium saturation decreases the free energy of the open state, favoring the association with proteins preferentially binding to that conformation and hindering the association with proteins preferentially binding to the closed state. Symmetrically, protein binding partners shift the conformation equilibrium of CaM towards their preferred state, which results in apparent affinity changes for calcium binding CaM.

**Fig 1:**
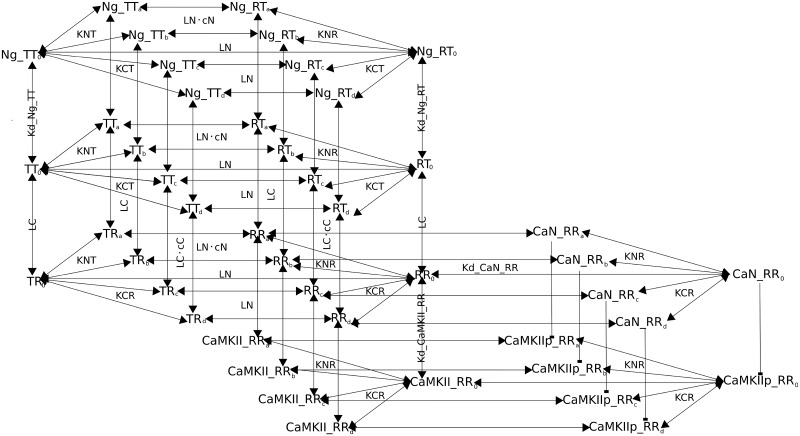
Reaction Diagram involving CaM and its binding partners. Binding interactions and state transitions involving calcium, CaM and CaM-binding proteins. For simplicity, only the binding of the first calcium ion is included. The reactions shown here are applied to all other calcium-bound forms, with any combination of filled calcium-binding sites. a and b represents calcium binding on CaM N lobe; c and d represent calcium binding on the C lobe. R indicates open lobes; T closed lobes. Dissociation coefficients for calcium at specific binding sites are specified, as well as state transition constants (ratios between opposite state transition rates). Identical dissociation constants for protein binding CaM are only written once. Parameter values are included in Table 1.

On top of the model developed previously by Lai *et al*. [25], We explicitly modeled CaM’s interaction with CaMKII, CaN and Ng. We detailed the autophosphorylation of CaMKII monomers. We modeled their dephosphorylation as if directly mediated by CaN, omitting the intermediate reactions involving DARPP-32/Inhibitor-1 and Protein Phosphatase 1. We also implemented reactions to maintain basal calcium concentration, as well as enabling calcium spikes at different frequencies, amplitudes, and duration. Reactions were primarily encoded in mass-action law kinetics except for the reactions depicting the calcium pump and CaMKII dephosphorylation. The model structure is partially illustrated in Fig 1, using no calcium or one calcium ion bound CaM as examples. The full model is written by the Systems Biology Markup Language (SBML) [48] in the supplementary material and has been deposited in BioModels [49] (accession number: MODEL1903010001).

First, we modified the hemiconcerted CaM model by Lai *et al*. 2015 [25], to allow the binding of calcium ions to affect equally, but in opposite directions, both transitions of CaM lobes between open and closed conformations. In other words, calcium binding not only speeds up the T to R state transition but also slows down the R to T state transition. We also removed the assumption that protein partners can bind to all possible conformations of CaM. Rather, we defined these bindings based on the described properties of each specific protein.

CaMKII monomer binds to CaM when both lobes are in open states (namely the “RR” conformation). This binding exposes the kinase domain of CaMKII monomer, allowing it to phosphorylate and to be phosphorylated by its neighbor monomers within the same hexamer ring if they are also in the active conformation [50]. We adapted and improved the approach used by Li *et al*. 2012 [10] to compute the probability of having an adjacent active monomer, at each time step, based on the proportion of active monomers in the whole system (CaM bound and/or phosphorylated). For more details, see the section CaMKII autophosphorylation below. We then used this probability to adjust the global autophosphorylation rate of CaMKII monomers. Once phosphorylated, CaMKII monomers have a higher affinity for CaM than their non-phosphorylated counterparts and remain active even upon CaM dissociation [51]. CaMKII monomers are dephosphorylated by Protein Phosphatase 1 (PP1). As CaN activates PP1 “linearly” through dephosphorylation of Thr34-phospho-DARPP-32, which then releases PP1 inhibition, we simply modeled a direct dephosphorylation of CaMKII monomers by CaN using Henry-Michaelis-Menten kinetics with total active CaN as the enzyme concentration.

CaN is a heterodimer containing a regulatory subunit (CaNB), and a catalytic subunit (CaNA) [52]. In our model, the sequential binding of four calcium ions to CaNB is required before CaNA can bind CaM, thereafter becoming active [53, 54]. CaNA binds to CaM when both CaM lobes are in the open state (“RR”).

As Ng interacts mostly with the C lobe, in this model we considered that it bound to CaM only when the C lobe was in the closed state, regardless of the N lobe conformation (“RT” and “TT”). As a consequence, the association of CaM with Ng does not prevent the transitions of the N lobe between T and R states. Moreover, due to the lack of relevant experimental data, we assumed that the binding of Ng on the C lobe did not exert any allosteric effect on those transitions.

To create a basal level of calcium (0.08 ⋅ 10^−6^ M), we implemented reactions to create constant influx and removal of ions, by mimicking a passive calcium input channel and a concentration-dependent calcium pump. Calcium input was encoded as a train of calcium spikes at varied frequencies. Each calcium spike was generated by adding a zero-order calcium creation during 8 ms (mimicking the opening of calcium channels), which elevated the free calcium concentration transiently to 0.8 ⋅ 10^−6^ M. The spike’s half-life time was about 30 ms, corresponding to the experimental observation [55]. For each free calcium spike, a total of 1926 calcium ions were injected in the model over an 8 ms period. All reactions occur in a single homogeneous compartment with a volume of 10^−15^ *L*.

### Estimation of equilibrium constants for CaM and calcium

The function of allosteric proteins is characterized by two types of parameters. The thermal equilibrium is quantified by *L*, the concentration ratio between the different states in the absence of any allosteric effector. In our case, CaM lobes exist under two states, closed (T), and open (R). And for each lobe, we have an allosteric constant *L* = [*T*]/[*R*]. Extremely large or extremely small *L* indicates a system for which the equilibrium is strongly biased towards a given conformation. In addition, for each allosteric effector, the ratio of their binding affinities for the two conformations of CaM, dubbed *c* for calcium (*cC* = *KCR*/*KCT*; *cN* = *KNR*/*KNT*) or *e* for Ng (*e* = *Kd*_Ng_*RT*/*Kd*_Ng_*TT* = 1), indicates if a ligand favors one conformation or the other. In addition, the allosteric parameter *c* reflects the influence of calcium binding on state transitions. A larger *c* indicates smaller effect, exerted by calcium binding, on state transitions, or *vice versa*.

We estimated the allosteric parameters for both N and C lobes by using experimental data obtained predominantly from full-length CaM, where intrinsic phenylalanine and tyrosine fluorescence were used for monitoring calcium saturation. We used three sets of steady-state experimental data: 1) CaM titrating CaMKII peptide with a saturating amount of calcium ions [6], 2) calcium saturation curves of the C lobe in the full-length CaM either without targets or in the presence of CaMKII peptide or full-length Ng [6, 26], 3) calcium saturation curve of the N lobe, in full-length CaM, without targets [6]. As the experimental data concerning the N lobe of CaM is relatively scarce, we also used a calcium titration curve of a truncated N lobe of CaM, that was locked in closed conformation [56].

To fully characterize the interactions between CaM and calcium, we thus have to estimate: 1) the binding affinities of calcium to the T state of CaM for sites on both lobes. Following Lai *et al*. [25], we further hypothesized that within each CaM lobe, the affinities of the two calcium-binding sites were the same and we had only two affinities to estimate (*KNT* and *KCT*), 2) the ratio between calcium affinity for the R and T states for both lobes (*cN* and *cC*) which we assumed to be equal for the two binding sites within each lobe as in Lai *et al*. 2015 [25], 3) the ratio of T state CaM to R state in the absence of calcium for both lobes (*LN* and *LC*), and 4) the affinity of CaM to the CaMKII peptide (*Kd*_CaMKIIpep_*RR*) and Ng (*Kd*_Ng_*TT* and *Kd*_Ng_*RT*).

As for the full-length CaMKII, CaMKII peptide used in the published experimental datasets binds to CaM when both lobes are in the R state. Ng only interacts with CaM’s C lobe and, as explained in the model structure section, *Kd*_Ng_*RT* = *Kd*_Ng_*TT*, i.e. Ng binding does not affect the state transitions of the N lobe and *vice versa*. The parameters remaining to be estimated are therefore reduced to: *KNT*, *KCT*, *cN*, *cC*, *LN*, *LC*, *Kd*_CaMKIIpep_*RR*, and *Kd*_Ng_*TT*.

Even with the assumptions described above, the amount of parameters to estimate is large and correlations may arise between them. Moreover, the experimental conditions used in these estimation procedures are highly diverse. Thus, we proceeded in several stages.

First, using calcium titration experiments of truncated N lobe locked in the closed conformation by disulfide crosslinks [56], we estimated the affinity between calcium and the N lobe in the T state to be *KNT* = 9.38 ⋅ 10^−5^ M (S1a and S1b Fig).

Using experimental measurements where CaM titrates CaMKII peptide in the presence of saturating amount of calcium ions [6], we then estimated the affinity between CaM and CaMKII peptide (*Kd*_CaMKIIpep_*RR*). Because of the high calcium concentration, we assumed that almost all CaM molecules were in the R state. This gave a value of *Kd*_CaMKIIpep_*RR* = 5.6 ⋅ 10^−9^ M (S1c and S1d Fig). This only provides an upper bound for the dissociation constant between CaMKII peptide and RR CaM, as not all CaM molecules are in the RR state, even with the highest calcium concentration. In fact, this value was reduced to 3.2 ⋅ 10^−10^ M during the subsequent stages of parameter estimation.

Estimating parameter values requires sampling values within some given ranges. We calculated the boundary values of KCT and cC by assuming that the observed calcium saturation levels of the C lobe, in the presence either of CaMKII peptide or of Ng [6, 26], reflected the calcium-binding affinities when all C lobes were locked in the R or T state respectively, resulting in *cC*′ = 0.0011 (*cC* = *KCR*/*KCT*) and *KCT*′ = 1.3 ⋅ 10^−5^ M (S1e to S1g Fig). This value of *cC*′ was used as the upper bound for estimating the real *cC*, as in reality, the CaMKII peptide is not capable of locking all C lobes in the R state. Similarly, *KCT*′ was used as the lower bound for *KCT*, since not all C lobes are locked in the T state by Ng, i.e. the actual affinity of the T state for calcium is lower.

Since *KCT* had the same order of magnitude as our estimated *KNT*, and calcium affinity for the C lobe should be higher than for the N lobe, the range for the real *KCT* value was narrow. Thus, to further reduce the number of parameters to be estimated, we simply assumed that, in the closed state, both C and N lobe shared the same calcium binding affinities, that is *KCT* = *KNT* = 9.38 ⋅ 10^−5^ M.

Finally, we estimated the remaining parameters: *cC*, *cN*, *LC*, *LN* and *Kd*_*Ng*_*TT* together using the full CaM model with steady state calcium titration curves [6, 26] and the boundary values mentioned above. As shown in S2 Fig, all parameters except *cC* and *LC* are identifiable. Fitting results are illustrated in S3 Fig. As Lai *et al*. [25], we found *cC* and *LC* highly correlated. Fitted their logarithms with a linear regression model provided a slope of -1.9 (S4 Fig). We therefore further refined these two parameters while estimating the rate constants.

### Estimations of kinetic rate constants

We estimated the affinity between open CaM (conformation “RR”) and CaN by using the CaN dose-response to CaM, in the presence of saturating calcium concentration [57]. We also estimated the association constant between the two proteins using stopped-flow kinetic data, where mutant CaM was labeled by Acrylodan (*CaM*(*C*75)_*ACR*_) [57]. The estimated affinity was *Kd*_CaN_*RR* = 3.2 ⋅ 10^−9^ M, with an association constant *kon*_CaN = 2.3 ⋅ 10^7^ M^-1^·s^-1^ (S5 Fig).

We directly used the dissociation constants of CaMKII and phospho-CaMKII from CaM taken from the literature (*koff*_CaMKII_*RR* = 1.1 s^-1^; *koff*_CaMKIIp_*RR* = 8.7 ⋅ 10^−5^ s^-1^) [58], and therefore only had to estimate their association rate constants. Based on previously published works [23, 26], we hypothesized that calcium binding to the N lobe was 100 times faster than to the C lobe, (*kon*_*N* = 100 × *kon*_*C*), and these did not depend on the conformation of the lobes, therefore reducing the estimation of 8 unknown association rates to only 1, *kon*_*CT*. Finally, we assumed that the base state-transition rates were the same for the two lobes, *k*_*TtoRC*0 = *k*_*TtoRN*0. The specific state transition rates, for all liganded populations, depend on the number of calcium bound to the CaM lobe, the protein partner bound, and the allosteric parameters estimated for this lobe. We also made use of the relationship between *cC* and *LC* described above, and only had to estimate *cC*.

We used stopped-flow fluorometry measurements of quin-2 fluorescence increase, after the addition of calcium-saturated CaM mix, in the presence of either CaMKII or phospho-CaMKII [31], and measurements of native tyrosine fluorescence decrease in the CaM C-terminal in the presence of EGTA, with or without Ng [26].

Most of the parameters estimated at this stage were identifiable, except the association constant between Ng and CaM *kon*_*Ng*, as well as the base state-transition rate *k*_*TtoRC*0 (S6 and S7 Figs). Thus, we chose the highest reasonable association constant for Ng and CaM (*kon*_Ng = 10^14^ M^-1^·s^-1^) and an average value for the transition rate among the range of values that all reached best fit (*k*_*T*2*RC*0 = 316227). The fitted results are illustrated in S8 Fig.

### CaMKII autophosphorylation

During simulations, we actively adjusted the global autophosphorylation rate of CaMKII monomers based on the total amount of active monomers, that are the phosphorylated and/or CaM bound monomers, and the likelihood of them having adjacent active monomers in pseudo hexamer rings, an updated approach from our previous work [10].

Briefly speaking, there are 7 types of hexamers, containing 0 to 6 active monomers. For each type, the possibilities of location for the active monomers are limited and we can easily compute the probability for an active monomer to have a neighbor also active. The main aspect of the procedure is to approximate the fraction of the different types of hexamers when the total number of active monomers is updated from the simulation. We achieved this by running, for every 1 percent increase of active monomers, 1000 independent random distributions of the active monomers to hexamers. For every increase of active monomers, we then computed the average occurrences of each type of hexamers and transformed them into the probability for a monomer to be distributed in a specific type of hexamer. We then multiplied the distributions of monomers with their corresponding probability of adjacent active neighbors. Finally, the summation of the above was used as the overall probability and the chance of having an active neighbor monomer for each percent increase in total active monomers. We fitted this data with a 5^th^ order polynomial function and embedded it into the model to adjust the autophosphorylation rate of CaMKII monomer (S9 Fig).

### Numerical simulations and analysis of results

All simulations, including timecourses with calcium spikes, were performed with COPASI [59], using the LSODA solver [60]. Parameter estimations were conducted using the SBPIPE package [61], combined with the “Particle swarm” optimization method (2000 iterations with a swarm size of 50). Parameters used for simulation of the model are listed in Table 1.

**Table 1:** List of parameters used in the model.

Estimated parameters for the N lobe (site: a,b)		Estimated parameters for the C lobe (site: c,d)	
**Allosteric parameters**:			
*cN*	0.01	*cC*	5 ⋅ 10^−5^
*LN*	299	*LC*	195865
**Dissociation constants of calcium from calcium lobes**:			
*KNT*	9.38 ⋅ 10^−5^ M	*KCT*	9.38 ⋅ 10^−5^ M
*KNR*	*KNT* × *cN* = 9.38 ⋅ 10^−7^ M	*KCR*	*KCT* × *cC* = 4.69 ⋅ 10^−9^ M
**Calcium binding rates**:			
*kon*_*N*	1.22 ⋅ 10^10^ M^−1^ ⋅ s^−1^	*kon*_*C*	1.22 ⋅ 10^8^ M^−1^ ⋅ s^−1^
**Calcium dissociation rates**:			
*koff*_*NT*	1.14 ⋅ 10^6^ s^−1^	*koff*_*CT*	1.14 ⋅ 10^4^ s^−1^
*koff*_*NR*	1.14 ⋅ 10^4^ s^−1^	*koff*_*CR*	0.6 s^−1^
**CaM state transition rates (0,1,2: number of calcium ions bound to that lobe)**:			
*k*_*TtoR*_*N*0	316227 s^−1^	*k*_*TtoR*_*C*0	316227 s^−1^
*k*_*RtoT*_*N*0	*k*_*TtoR*_*N*0 ⋅ *LN* ≈ 9.46 ⋅ 10^7^ s^−1^	*k*_*RtoT*_*C*0	*k*_*TtoR*_*C*0 ⋅ *LC* ≈ 6.2 ⋅ 10^10^ s^−1^
*k*_*TtoR*_*N*1	$k\_TtoR\_N0/\sqrt{cN}\approx 3.16\cdot 10^{6}\;s^{-1}$	*k*_*TtoR*_*C*1	$k\_TtoR\_C0/\sqrt{cC}\approx 4.47\cdot 10^{7}\;s^{-1}$
*k*_*RtoT*_*N*1	$k\_RtoT\_N0\cdot \sqrt{cN}\approx 9.46\cdot 10^{6}\;s^{-1}$	*k*_*RtoT*_*C*1	$k\_RtoT\_C0\cdot \sqrt{cC}\approx 4.38\cdot 10^{8}\;s^{-1}$
*k*_*TtoR*_*N*2	*k*_*TtoR*_*N*0/*cN* ≈ 3.16 ⋅ 10^7^ s^−1^ s^−1^	*k*_*TtoR*_*C*2	*k*_*TtoR*_*C*0/*cC* ≈ 6.32 ⋅ 10^9^ s^−1^
*k*_*RtoT*_*N*2	*k*_*RtoT*_*N*0 ⋅ *cN* ≈ 9.46 ⋅ 10^5^ s^−1^	*k*_*R*2*T*_*C*2	*k*_*R*2*T*_*C*0 ⋅ *cC* ≈ 3.1 ⋅ 10^6^ s^−1^
**Parameters relating to CaM-binding substrates**:			
*k*_CaMKII_*autop*	adjusted_coefficient × 6.3 s^−1^	This is adjusted at each time step during simulation. Base rate is from [62].	
*koff*_CaMKII_*RR*	1.1 s^−1^	CaMKII’s dissociation rate from active CaM [58].	
*kon*_CaMKII_*RR*	2.87 ⋅ 10^6^ M^−1^ ⋅ s^−1^	Estimated CaMKII’s binding rate to active CaM	
*koff*_CaMKIIp_*RR*	8.7 ⋅ 10^−5^ s^−1^	Thr286-phospho-CaMKII’s dissociation rate from active CaM [58]	
*kon*_CaMKIIp_*RR*	3.85 ⋅ 10^7^ M^−1^ ⋅ s^−1^	Estimated Thr286-phospho-CaMKII’s binding rate to active CaM	
*kon*_CaN_Ca1, 2, 3, 4	2 ⋅ 10^7^ M^−1^ ⋅ s^−1^	CaN B’s binding rate to Ca [10]	
*koff*_CaN_Ca	0.0092 s^−1^	[10]	
*koff*_CaN_Ca_Ca	0.0312 s^−1^	[10]	
*koff*_CaN_Ca2_Ca	0.352 s^−1^	[10]	
*koff*_CaN_Ca3_Ca	0.9 s^−1^	[10]	
*kon*_CaN_*RR*	2.3 ⋅ 10^7^ M^−1^ ⋅ s^−1^	Estimated CaN’s binding rate to activated CaM	
*Kd*_CaN_*RR*	3.26 ⋅ 10^−9^ M	Estimated CaN’s dissociation constant from activated CaM	
*kon*_Ng_*RT* *kon*_Ng_*TT*	1 ⋅ 10^14^ M^−1^ ⋅ s^−1^	Estimated Ng’s binding rate to CaM when C lobe is closed	
*Kd*_Ng_*RT* *Kd*_Ng_*TT*	1.2 ⋅ 10^−6^ M	Estimated Ng’s dissociation constant from CaM, when C lobe is closed	
*kcat*_CaN_CaMKIIp	0.8 s^−1^	CaN’s catalytic efficiency on dephosphorylating CaMKII Used in this paper	
*Km*_CaN_CaMKIIp	2 ⋅ 10^−5^ M	CaN’s Michaelis-Menten constant on dephosphorylating CaMKII Used in this paper	
**Concentrations used in the model (unless specified)**:			
basal *Ca* ^2+^	8 ⋅ 10^−8^ M [63]	*CaM*	4 ⋅ 10^−5^ M [64]
*CaN*	8 ⋅ 10^−6^ M [64]	*CaMKII*	8 ⋅ 10^−5^ M [64]
*Ng*	4 ⋅ 10^−5^ M [64]	compartment volume	1 ⋅ 10^−15^ L
**Other Parameters)**:			
*Ca* ^2+^ leak	→ *Ca* ^2+^	3.85 ⋅ 10^−6^ M ⋅ s^−1^	
*Ca* ^2+^ pump	*Ca* ^2+^ →	*Km* = 2 ⋅ 10^−6^ M	*Vmax* = 1 ⋅ 10^−4^ M ⋅ s^−1^

For parameter estimations, please see the method and supplementary sections. R: open; T: closed. When combined, the first letter indicates the state of CaM’s N lobe while the second one indicates the state of the C lobe. Since *kon*, *koff*, and *Kd* are linked (*Kd* = *koff*/*kon*), only two of these three are displayed, depending on which ones were actually estimated. For parameters concerning sequential dissociation of Ca^2+^ ions from CaN, the first “Ca” indicates the number of Ca^2+^ ions bound to CaN, the second “Ca” shows the one dissociating from CaN.

The frequency of calcium spikes was directly encoded in the model together with the number of spikes, to control the total duration of calcium inputs. We ran the simulations on a computing cluster, using Python’s *ElementTree* package to automatically modify the frequency parameter in CopasiML files.

Each simulation started with a 5000 s equilibrium—with output interval size set at 1 s, followed by the simulation of 300 calcium spikes at frequencies ranging from 0.1 Hz to 100 Hz, with output interval size set at 0.0001 s. We recorded the evolution of all protein active forms during 3000 s.

In order to evaluate CaMKII and CaN’s effects on synaptic plasticity, we measured the “activated area” [10], which was obtained by integrating, over the 3000 s, the product of their relative activation (concentration of active proteins over total concentration) above basal activities (subtracting basal levels), by their catalytic constants on *α*-amino-3-hydroxy-5-methyl-4-isoxazolepropionic acid (AMPA) receptor GluR1 subunit. The BCM-like curve [65], useful to characterize bidirectional plasticity, was obtained by subtracting the activated area of CaN from that of CaMKII for each calcium spike frequency.

## Results

### Ng sequesters closed-state CaM

**Fig 2:**
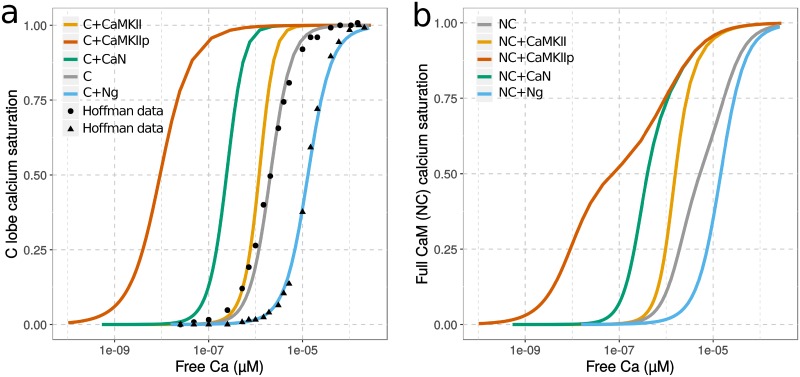
Saturation curves of CaM by calcium. Values were obtained by scanning a range of initial calcium concentrations and running simulations until steady-state is reached, using initial conditions described in Hoffman *et al*. 2014 [26]. a) shows only the saturation of C lobe (within the context of the entire CaM) while b) shows the whole CaM, without any substrates (grey), with Ng (blue), non-phosphorylated CaMKII (orange), CaN (green) or Thr286-phospho-CaMKII (red). The x-axis shows the *free* concentration of calcium and not the initial concentration used for the scan. [CaM] = 5 μM; [Ng/CaMKII/CaMKIIp/CaN] = 50 μM. Solid line: simulation results; dots: experimental observations.

We first studied how CaM-binding proteins can affect CaM’s apparent affinity for calcium at steady states. As shown in Fig 2, and in accord with previous experiments [6, 26] and modeling [25], the presence of Ng shifts the calcium-saturation curve of CaM’s C lobe towards higher calcium concentration ranges, reducing the apparent affinity for calcium. This indicates that Ng traps the C lobe in the closed, low-affinity, conformational state, and therefore hinders calcium binding, in particular at low calcium concentrations. Conversely, CaMKII and CaN promote calcium binding to the C lobe, increasing the apparent affinity, through their preferential binding to the open, high-affinity conformation (of both lobes).

Calcium saturation curves for full CaM show similar trends as for the C lobe (Fig 2), but they also indicate CaM’s lobe differences in terms of calcium binding. The allosteric parameters we estimated predict that the N lobe is more flexible and has greater potential to undergo spontaneous conformational transitions than the C lobe (*LN* closer to 1 than *LC*). However, the binding of calcium has less influence on the conformation of the N lobe, because of the smaller ratio between the affinities for calcium of the two conformations in the N lobe compared with the C lobe (*cN* closer to 1 than *cC*). The saturation curve of full CaM in the absence of protein binding partners stretches across a wider range of calcium concentration than the C lobe, showing a larger contribution of the C lobe at lower calcium concentrations. As in our model Ng only interacts with the C lobe, it has little effect on calcium binding to the N lobe. As a consequence, the shift in the saturation curve is mostly seen at lower calcium concentrations. Because both N and C lobes wrap around CaMKII and CaN, the presence of these proteins greatly enhances calcium binding not only to the C lobe but also to the N lobe. However, the N lobe’s calcium affinity is less increased by the conformational change to the open state. Although having the highest affinity towards CaM, phospho-CaMKII cannot shift N lobe’s affinity much compared to non-phospho CaMKII.

**Fig 3:**
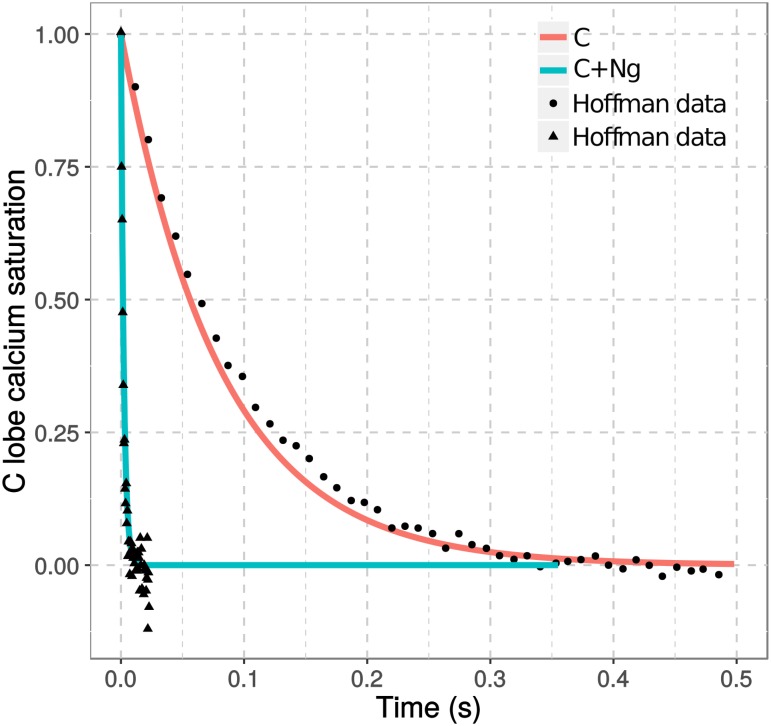
Kinetics of calcium dissociation from CaM. Calcium dissociation from the C lobe of CaM was simulated upon mixing calcium chelator EGTA (10^−2^ M) after the model simulation reached steady states with pre-mixed CaM (10 μM) and calcium (100 μM) with (cyan) or without (salmon) Ng (50 μM). Solid line: simulation results; dots: experimental observations [26].

In line with the observations above, timecourse simulations show that the presence of Ng speeds up calcium dissociation from CaM’s C lobe (Fig 3), in agreement with stopped-flow fluorometry experimental observations [26]. The steady-state and kinetic results validate our model assumptions and parameters. They also confirm well established functions of Ng: Ng decreases calcium binding to CaM at steady states; Ng increases calcium dissociation from CaM when free calcium concentration is decreased. Both observations arise from the preferential binding of Ng to a conformation of CaM’s C lobe with a low affinity for calcium.

### Ng shifts CaM activation towards high-frequency calcium spikes

**Fig 4:**
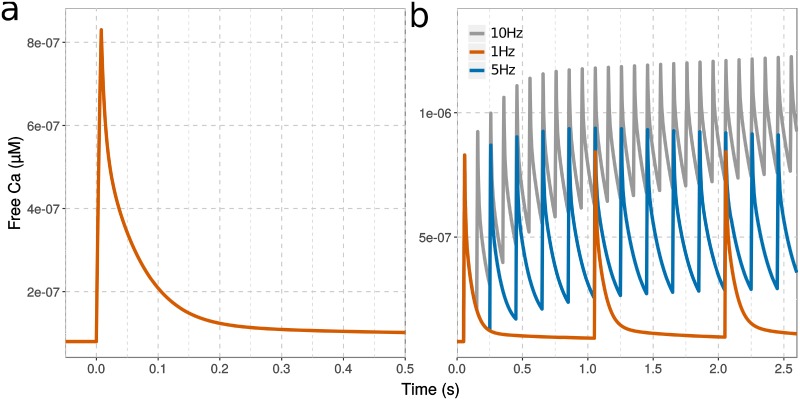
Free calcium concentration at various input frequencies. Intracellular free calcium elevation simulated with a single calcium input (a) or a train of inputs (b) at 1 Hz (red), 5 Hz (blue) and 10 Hz (grey). Each calcium spike represent the addition of 1926 ions over 8 ms.

Post-synaptic stimulation triggers calcium spikes. During one spike, free calcium concentration transiently reaches a near-micromolar level in a few milliseconds, and subsequently declines in about 200 milliseconds, due to calcium pumps and calcium-binding proteins [55]. Therefore, rather than looking at the responses of CaM to steady elevations of calcium concentration, we must look at its responses to calcium spikes at different frequencies (Fig 4).

**Fig 5:**
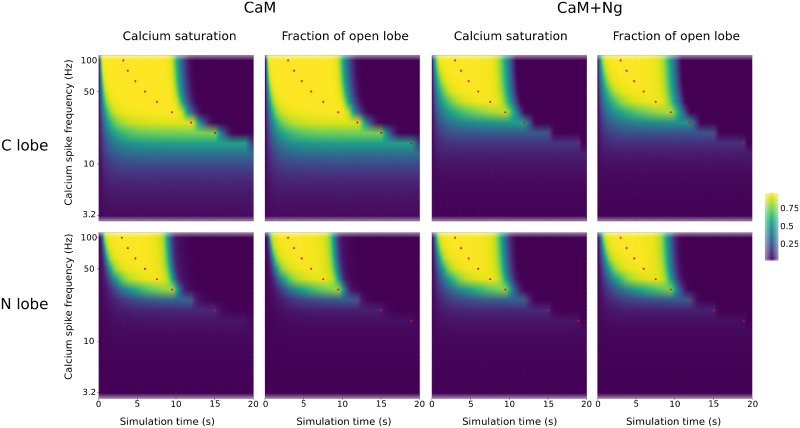
Calcium binding and CaM conformational changes in response to calcium input frequencies. Calcium binding CaM was followed during simulations using trains of 300 spikes at different frequencies. Plots show calcium saturation levels at the C and N lobe and the corresponding CaM conformational changes, with CaM on its own, or in the presence of Ng. Red dots represent the end of calcium stimulation. [CaM]_tot_ = 40 μM, [Ng]_tot_ = 40 μM, basal [Ca] = 0.08 μM.

First we simulated the CaM model without protein partners, using a fixed total calcium input applied at varied spike frequencies. The results are in line with the behavior of our previous fully concerted model [10], and show that both CaM lobes respond differently to different calcium spike frequencies. At high spike frequencies, both lobes stay in the open state for a duration longer than calcium inputs (red dots in Fig 5 indicate the end of calcium stimulation). However, the N and C lobes display different sensitivities towards calcium spike frequencies. The C lobe opens at lower frequencies, and for a longer duration, whereas the N lobe requires much higher spike frequencies to switch to the open conformation. In both lobes, the stabilization of the open conformation follows the profiles of calcium binding.

When Ng was added to the model, the range of frequencies able to open most C lobes narrows towards high values (Fig 5). The duration of these openings is also shorter than when Ng is absent. Unsurprisingly, no change was observed for the N lobe (Fig 5). Overall, the opening patterns of the C lobe in presence of Ng resemble those of the N lobe, restricting the opening to high calcium input frequencies.

**Fig 6:**
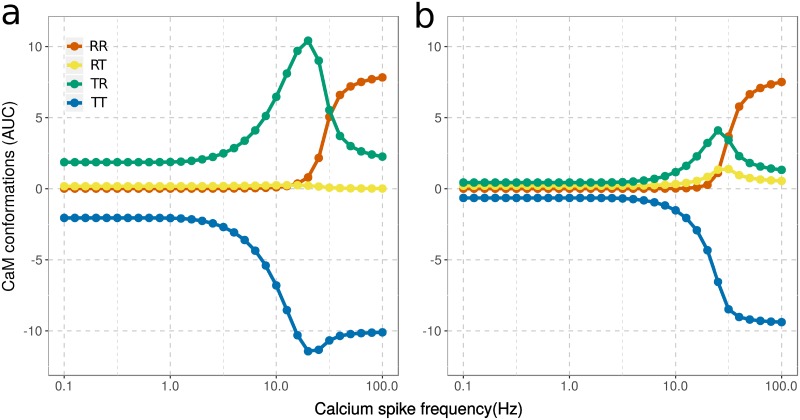
AUCs of CaM conformations in response to calcium input frequencies. Conformational changes of CaM (40 μM) without Ng (a) or with Ng ([Ng]_tot_ = 40 μM) (b). Basal activities were subtracted before AUCs were calculated.

To better quantify CaM’s responses to the frequencies of calcium input, we calculated the Area Under the Curve (AUC), that is the integral of the increased/decreased fraction of CaM in the open/closed state (above/below basal level), over the entire simulation (see Methods). We calculated these AUCs for all the possible conformations of CaM lobes: RR, RT, TR and TT (where the first letter represents the state of the N lobe and the second represents the state of the C lobe. We then plotted them against calcium spike frequencies (Fig 6).

In the absence of Ng, CaM’s C lobe is very sensitive to calcium spikes at low frequencies. The TR conformation is increased above its basal level (AUC>0) by calcium stimulation as low as 0.1 Hz (Fig 6a). The TR conformation increases steeply around 3 Hz, peaks at 20 Hz, then declines while the N lobe of the same CaM opens (increase of the RR conformation). The RT conformation does not increase in response to calcium spikes. There is almost one order of magnitude between the frequencies at which the C and N lobe open prominently (Fig 6a).

Ng not only decreases the C lobe opening in response to low frequencies but also shifts it towards higher frequencies. At about 10 Hz, both the TR and RT state start to increase and peak around 25 Hz, indicating the opening of C and N lobes separately within two populations of CaM molecules (Fig 6b). It seems that the N lobe can compete with the C lobe for calcium binding to stabilize its open conformation only in the presence of Ng (Fig 6a). Calcium binding to the N lobe is 100 times faster than its binding to the C lobe. However, the rate of dissociation from the N lobe is also extremely high (corresponding to a lower affinity), which makes the N lobe less efficient than the C lobe for calcium binding. The rates of transition from the closed to open conformation, when no calcium ion is bound, are the same for both lobes. As a result, at these intermediate frequencies, Ng facilitates the opening of the N lobe by decreasing the opening of the C lobe and thus its binding of calcium. At high spike frequencies, the opening of both lobes (RR state) follows almost the same profile as when Ng is absent and reaches the same activity level at 100 Hz regardless of Ng’s presence (Fig 6b).

Therefore, Ng does not prevent CaM activation by calcium spikes. Instead, Ng synchronizes both lobes to respond towards higher calcium spike frequencies, hence narrowing the frequency range at which CaM responses.

### Ng facilitates CaMKII activation at high calcium spike frequencies

We then investigated Ng’s role in regulating the activation of CaMKII and CaN by CaM. We first added only CaMKII and CaN in the model (without Ng), as well as the inhibition of CaMKII autophosphorylation by CaN (see Materials and methods). In all the subsequent simulations, the concentration of CaM is smaller than the total concentration of its binding proteins. As above, we simulated trains of calcium spikes at various frequencies but resulting in a fixed additional amount of calcium ions. To compute their activity on phosphorylation of AMPA receptor subunit GluR1, we multiplied the fraction of active CaMKII and CaN by their respective catalytic constants (kcat).

**Fig 7:**
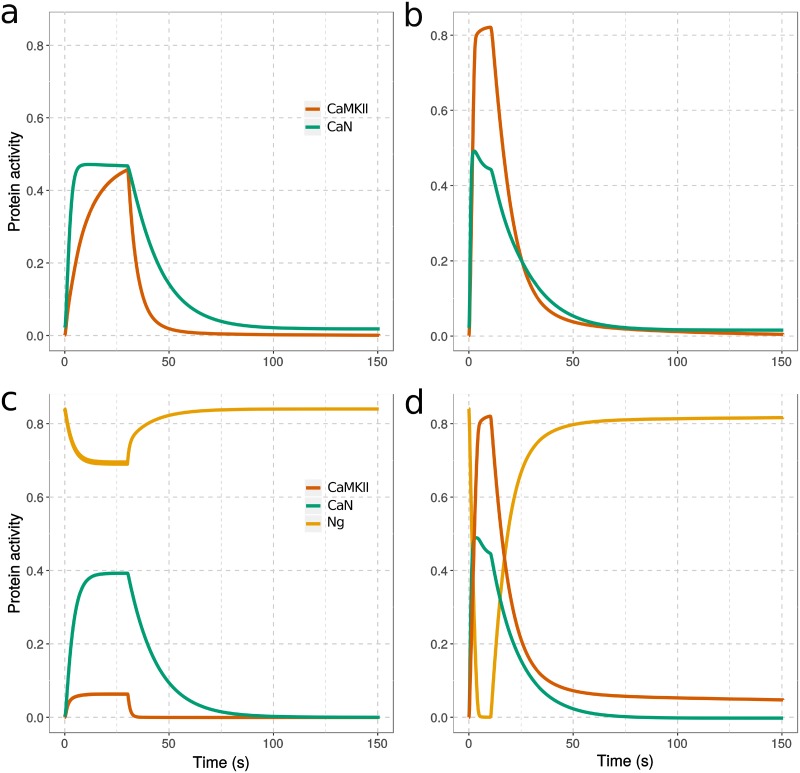
Change of protein activity in response to calcium spikes. Simulations in absence (a,b) or presence (c,d) of Ng, from equilibrium and during stimulation by 300 calcium spikes at 10 Hz (a,c) and 30 Hz (b,d). The protein activities were defined as follow: fraction of CaMKII monomers bound to CaM and/or phosphorylated multiplied by CaMKII kcat for GluR1, fraction of CaN bound to CaM multiplied by CaNA kcat for GluR1, fraction of Ng bound to CaM. [CaM]_tot_ = 40 μM, [CaMKII]_tot_ = 80 μM, [CaN]_tot_ = 8 μM, and [Ng]_tot_ = 40 μM; *kcat*_CaMKII_ = 2 s^-1^, *kcat*_CaN_ = 0.5 s^-1^. All the concentrations of perspective CaM binding proteins are assigned according to the proteomic study in the hippocampus CA1 region [64].

Fig 7 shows example timecourses of protein activity changes following 300 calcium-spike stimulations at 10 Hz or 30 Hz. In the absence of Ng, resting calcium level opens a small proportion of CaM, resulting in higher basal CaN activity than CaMKII. With a 10 Hz calcium stimulation, the opening of CaM leads to a fast and strong elevation of CaN, accompanied by a gentler rise of CaMKII activity, with CaN and CaMKII activities reaching to the same level (Fig 7a). At 30 Hz, the acute elevation of calcium gives rises to sharp and prominent increases of both CaMKII and CaN activities, and the activity of CaMKII overcomes that of CaN. However, the activation of CaMKII decays very quickly to its resting level, potentially due to the slower deactivation and high basal activity of CaN, which prevents the onset of CaMKII autophosphorylation (Fig 7b).

When Ng is present in the model, CaN basal activity is lower at resting calcium concentration, indicating decreased open CaM because of Ng binding. Calcium inputs at both frequencies raise Ca-saturation of CaM, stabilizing open CaM free of any protein partner. This triggers an equilibrium shift (reduced closed CaM free of protein partners) and a release of CaM from Ng. At 10 Hz, a small proportion of CaM is released from Ng, inducing a strong activation of CaN, but a much weaker activation of CaMKII (Fig 7c). At 30 Hz, Ng releases almost all CaM. This transient but strong CaM release robustly increases CaMKII and CaN activity. At both frequencies, the decay of CaN activity is faster than when Ng is absent. The most striking difference is in the way CaMKII activity decays. A significant proportion of CaMKII monomers remain active, and autophosphorylated, for more than 10 minutes, which is far beyond the total duration of calcium stimulation (Fig 7d and S10 Fig). These results indicate an multi-phasic regulation of CaMKII activity by Ng. At an intermediate spike frequency (10 Hz), Ng restrains CaMKII activation; at a high spike frequency (30 Hz), Ng promotes it.

**Fig 8:**
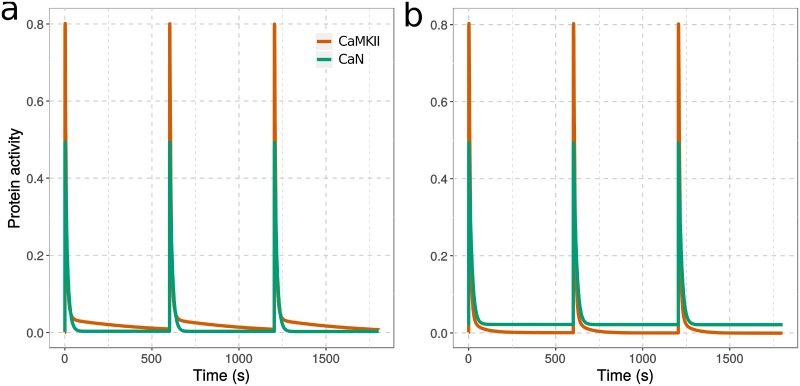
CaMKII and CaN activation in response to repeated high-frequency calcium spikes. Computational models were simulated, with (a) or without (b) Ng, by 300 calcium spikes organized into three 100-spike discrete bursts at 100 Hz each, separated by 10 min intervals. Each calcium input was as described in Fig 3. Both protein activities were normalized to their total concentration, then multiplied by their catalytic constant for GluR1. [CaM] = 40 μM, [Ng] = 40 μM, [CaN] = 8 μM, [CaMKII] = 80 μM; *kcat*_CaMKII_ = 2 s^-1^; *kcat*_CaN_ = 0.5^-1^.

In most LTP-induction protocols, the high-frequency stimulation is not applied continuously but split into discrete bursts. We verified the observation presented above by stimulating the model with three 100 Hz, 100-spike bursts, separated by 10-minute intervals [15]. As shown in Fig 8, we obtained results similar to a setup featuring a continuous 300-spike stimulation (S11 Fig). When Ng is present (Fig 8a), a significant proportion of CaMKII monomers retain their activities for about 10 minutes after the end of each burst. Whereas in the absence of Ng (Fig 8b), the elevated basal CaN prevents such a long-lasting CaMKII activation, resulting in a more transient CaMKII activity. Interestingly, with such a discrete-bursts stimulation, the overall CaMKII activity (integrated over time) is increased by nearly 20% compared with continuous 300-spike stimulation.

**Fig 9:**
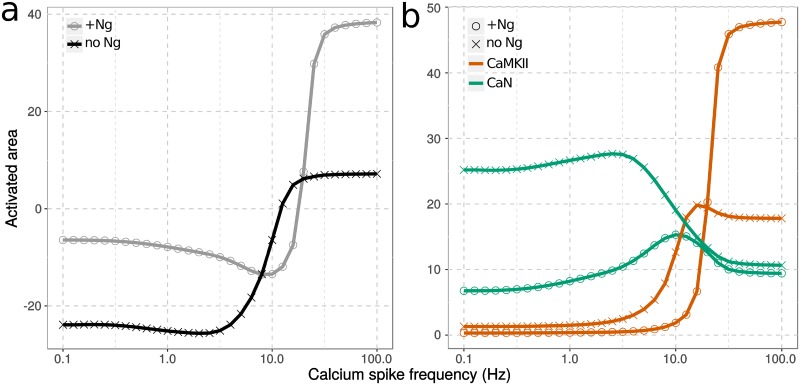
Activity of CaMKII and CaN in response to calcium spike frequencies. Protein activities, as described in Fig 7, were integrated over time. The effect on synaptic plasticity was calculated by subtracting CaN’s activated area from CaMKII (a). Each protein’s activated area was also plotted as a function of calcium input frequency (b). Models were stimulated either with (grey circle) or without Ng (black cross). All protein concentrations were as described in Fig 7 and in Table 1.

We systematically explored 30 frequencies, ranging from 0.1 to 100 Hz. To represent the combined effect of CaMKII and CaN on GluR1, thus their impact on synaptic plasticity, we subtracted their “activated area”, following a continuous 300-spike stimulation at each frequency. The “activated area” is an integration of the activity change of an enzyme (above basal level) along time, after the calcium stimulation of a specific frequency. It was calculated based on the timecourse result, such as examples in Fig 7. For each curve in Fig 9a, negative values indicate that CaN activation overcomes CaMKII activation, thus facilitating LTD. Whereas positive values show that CaMKII activation overcomes CaN activation, thus facilitating LTP. Both curves, with (circle) and without (cross) Ng, show triphasic responses, reproducing the classical BCM curve, with predominant CaN activity at low calcium-spike frequencies and overriding CaMKII activity at high spike frequencies. However, the presence of Ng results in an upright shift of the frequency-response curve, indicating the hindered onset of LTD, increased frequency for LTP onset and lifted CaMKII responses at high spike frequencies.

The difference of responses in the presence and the absence of Ng is explained by its different effects on the “activated area” of CaN (green) and CaMKII (red), that vary according to the frequency (Fig 9b). Because of CaN’s relatively low concentration and its higher affinity for CaM than non-phospho CaMKII monomers, CaN becomes active even at low spike frequencies. This strongly inhibits CaMKII autophosphorylation. Despite this constant presence of phosphatase activity, both CaN and CaMKII activity change with frequency, including in the presence of Ng. Thus, Ng regulates the activation of CaMKII and CaN, modulating synaptic plasticity, in three phases: At low spike frequencies, Ng hinders the activation of both proteins, albeit with a stronger effect on CaN, thus inhibiting LTD. As the spike frequency increases, CaN activity rises to induce LTD. The declining phase of CaN activity corresponds to the increasing CaMKII activity, marking the transition from LTD to LTP. The presence of Ng shifts this transition to higher frequencies, therefore increasing the frequency required for LTP induction. At high spike frequencies, Ng predominantly facilitates CaMKII autophosphorylation, thus promoting LTP. As Ng’s regulatory role in synaptic plasticity and CaMKII activation depends on calcium-spike frequencies, and intracellular patterns of calcium elevation vary dependent on the LTP-induction protocols and the specific cellular environment (e.g. the number of receptors), Ng’s non-monotonic regulation might provide a clue to opposing experimental observations in Ng knock-out mice. Ng raises the threshold frequency to activate CaMKII. However, once the threshold is crossed, Ng facilitates its activation.

The fact that sustained CaMKII activity at high calcium spike frequencies was observed only in the presence of Ng was intriguing. To simulate the autophosphorylation of CaMKII monomers, we continuously adjusted the phosphorylation rate based on the total number of active CaMKII monomers (CaM bound and/or phosphorylated), and their random distributions within pseudo hexamer rings. Therefore, to initiate the autophosphorylation of CaMKII, a large number of CaM molecules need to bind to CaMKII, to ensure adjacent neighbor monomers are active within hexamer rings (see Method for more details). If the only function of Ng was to buffer CaM and preclude it from opening, it would not facilitate CaMKII activation, given that the concentration of CaM is already a limiting factor [10]. In the following section, we shed light on the mechanisms underlying Ng’s positive regulation on CaMKII activation at high calcium-spike frequencies.

### Ng facilitates CaMKII activation by suppressing the activity of CaN

Ng’s positive impact on CaMKII activation at high-frequency calcium stimulation has been observed experimentally [15, 19]. However, no underlying mechanism was suggested. Sustained CaMKII activation is due to its autophosphorylation, and CaN regulates its dephosphorylation via PP1. We thus hypothesized that CaN might play an important role in mediating Ng’s effect on CaMKII activation.

The frequency-response plot (Fig 9b) shows that the presence of Ng causes a significant change in the plateau activity of CaMKII at high spike frequencies, but a fairly small change of CaN activity. It is therefore difficult to see how such a small change could have an important effect on CaMKII autophosphorylation. However, the phosphorylation of CaMKII is regulated not only by the dynamic elevation of CaN following calcium spikes that is shown in Fig 9b, but also by CaN’s basal activity which is elevated in the absence of Ng (Fig 7a and 7b). This increased basal activity causes a greater change of CaN’s activated area without affecting the CaMKII curve (as shown on S12 Fig, where CaN’s frequency dependent curve is further increased by 54 units). Thus, at high spike frequencies, CaN activity is higher than CaMKII activity in the absence of Ng. However, in the presence of Ng, the activity of CaN is lower than CaMKII. This is reflected in the difference of “activated area” curves in S12 Fig.

**Fig 10:**
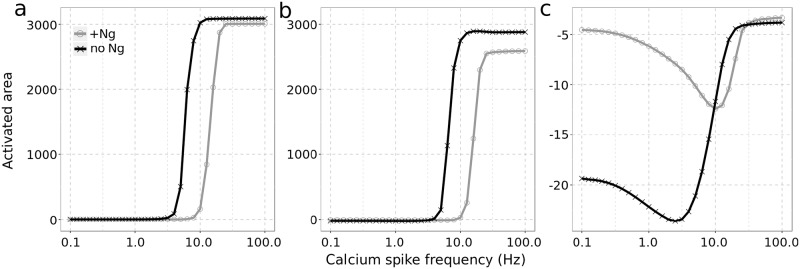
CaN mediates Ng’s effect on CaMKII activation. Models were simulated without CaN (a) or without CaN’s phosphatase activity (*kcat* = 0)(b) or with double amount of CaN (c). The combined response of CaMKII and CaN to calcium spike frequencies were quantified as described in Fig 9: with Ng (grey circle) and without (black cross). Simulation conditions and concentrations are as described in Fig 7. Double amount of CaN equals to 16 μM.

To further assess CaN’s role, we removed it from the model and re-simulated the various frequencies of calcium spikes, with and without Ng. As shown in Fig 10a, removing CaN dramatically increases CaMKII activity at high calcium-spike frequencies regardless of Ng’s presence. Moreover, CaMKII reaches similar maximal activity in both situations. As the presence of Ng does not increase CaMKII activity at high calcium-spike frequencies (see grey curve on Fig 10a), the previously observed positive impact of Ng on CaMKII activation (Fig 9) is completely lost. The key impact of removing Ng is a shift of the BCM-like curve towards lower spike frequencies, which indicates a negative effect of Ng on CaMKII in the absence of CaN.

To address whether it is CaN phosphatase activity or its high-affinity binding to CaM which mediates Ng’s effect on CaMKII activation, we simulated the model after removing CaN’s phosphatase activity (*kcat* = 0), keeping its concentration at the value used to generate Fig 9 (Table 1). Without CaN phosphatase activity, the presence of Ng decreases CaMKII activation at high spike frequencies (Fig 10b), showing that it retain only an inhibitory role. Therefore, Ng facilitates CaMKII activation by suppressing the phosphatase activity of CaN. Furthermore, CaN’s central role on mediating Ng’s effect cannot be replaced by any other high-affinity CaM-binding proteins.

We then doubled CaN concentration, to twice its detected level in the hippocampus CA1 region [64], while keeping its phosphatase activity (Fig 10c). At low spike frequencies, CaN activity suppresses CaMKII as seen above with normal CaN concentration. However, at high frequencies CaMKII activity can no longer overturn CaN activation, resulting in abolished LTP induction regardless of Ng’s presence. This indicates that high CaN concentration suppresses the positive effect of Ng on CaMKII activation and LTP induction at high frequency stimulations. To summarize, when there is not enough CaN, CaMKII activation is excessive and when there is too much CaN, CaMKII activation is insufficient. In both cases, Ng exerts no positive effect on CaMKII activation.

**Fig 11:**
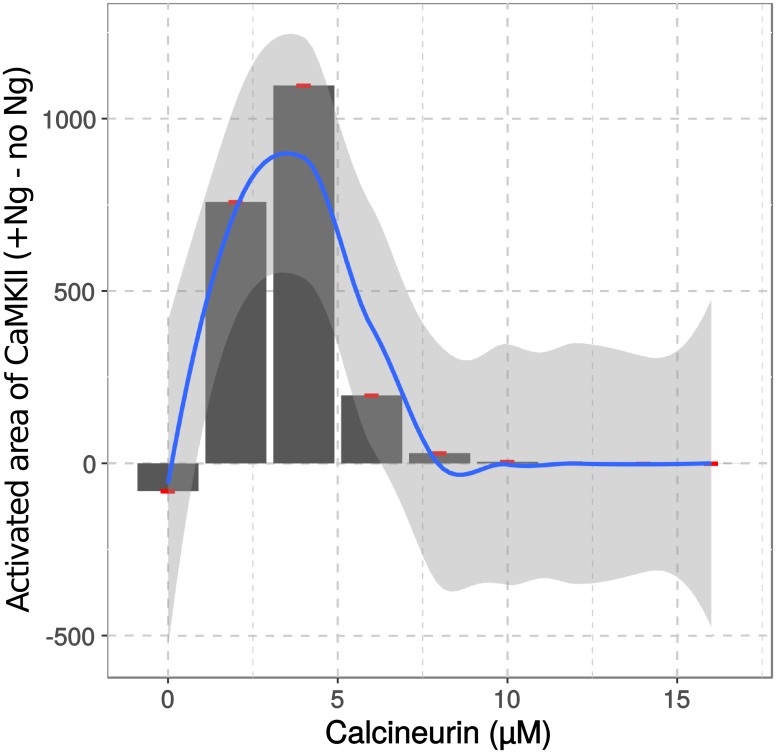
CaN concentration affects Ng regulation of CaMKII activation. Different CaN concentrations were applied in the presence and absence of Ng. The difference between CaMKII’s activated areas reached in response to a 100 Hz calcium spike frequency in presence and absence of Ng were plotted against CaN concentration. The relationship was further fitted into a polynomial function (method loess() from ggplot2) and plotted as the blue line. Red points are the values coming from our simulations. Shaded light grey area represents the 95% confidence interval for the fitting.

We tested other CaN concentrations and plotted the differences of CaMKII activation reached at a high calcium spike frequency (100 Hz), in the presence and absence of Ng. Fig 11 shows a nonlinear relationship between the concentration of CaN and the effect of Ng on CaMKII activation. It seems that with those concentrations of Ng, CaM, and CaMKII, Ng displays the highest positive effect on CaMKII activation when CaN concentration is about 4 micromolar. Reducing or increasing CaN concentration weakens Ng’s impact on CaMKII.

These results suggest that a key role for CaN is to control how Ng affects CaMKII activation at higher calcium spike frequencies. The presence of Ng facilitates a prompt release of large quantities of CaM at specific spike frequencies. This facilitates the onset of CaMKII activation and autophosphorylation, allowing it to overcome CaN’s negative feedback. Removal or over-expression of CaN eliminates Ng’s positive regulation on CaMKII, reducing its role to merely a CaM buffer.

### The concentration of Ng impacts LTP induction

Previous research showed that Ng constantly moves in and out of the PSD. Its function as a regulator of synaptic plasticity may partly rely on its recruitment of CaM in the PSD [66]. While this is certainly an important aspect of Ng’s function, modeling Ng and CaM translocation in and out the PSD may blur other Ng’s impacts on plasticity. We have already shown that the concentration of CaM is limited in the PSD and increasing it has a positive effect on LTP induction [10].

**Fig 12:**
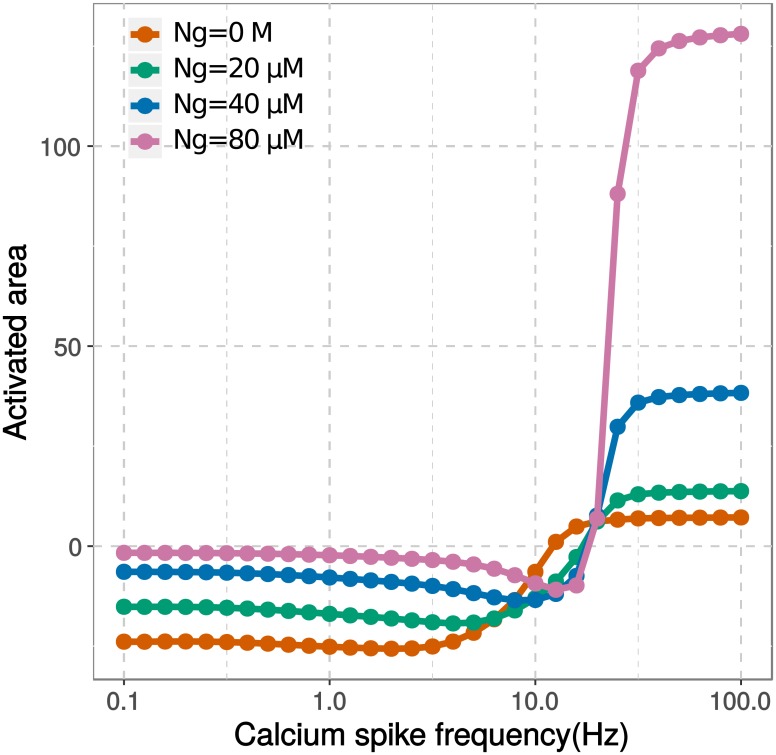
Ng concentration affects synaptic plasticity. Computational models with various Ng concentrations were stimulated by 300 calcium spikes at various frequencies. The concentrations of proteins, except Ng, were listed in Table 1 and remain unchanged. In particular, the concentration of CaM is 40 μM. The activated areas of CaMKII and CaN were calculated and their net effects on AMPA receptor phosphorylation were calculated as described in Fig 7, and plotted as a function of calcium spike frequency.

The model presented here only incorporated the binding of CaM by Ng in a homogeneous compartment. However, we showed that increasing the concentration of Ng, while keeping the concentration of CaM the same, has a positive effect on LTP induction. As shown in Fig 12, a higher Ng concentration results in a higher response from CaMKII over CaN, with a steeper transition from LTD to LTP. Despite a relative shift toward CaMKII at all frequencies, the transition between CaN and CaMKII (Θ_*m*_) occurs at higher frequencies when the concentration of Ng increases. Furthermore, the LTD response deepens when the concentration of Ng decreases whereas the LTP response strengthens when the concentration of Ng increases. Therefore, the synaptic response brought forward by Ng’s translocation may lie in between these lines with a broader dynamic range across responses of LTD and LTP.

## Discussion

CaM regulates synaptic plasticity via its binding partners [26, 31, 67], some of which have well-known functions while other non-catalytic binding proteins’ roles are less clear. One such protein is Ng, a highly expressed brain protein that carries a CaM-binding IQ domain [68, 69].

Ng primarily interacts with the closed C-lobe of CaM [13, 26], and is considered to buffer CaM [11, 12, 69, 70]. Despite many studies of its function in regulating synaptic plasticity, its precise role is still questioned. Several *in vivo* experiments showed that Ng facilitates CaMKII activation during LTP induction protocols [16, 19], and it is not a CaM buffer because CaMKII activation requires a significant amount of open CaM. To confuse matters further, the knock-out of Ng in mouse can either facilitate or inhibit LTP induction [14–19].

By setting up a mechanistic model of CaM and incorporating conformational changes of its two lobes, we explored the potential roles Ng plays in regulating bidirectional synaptic plasticity mediated by CaMKII and CaN.

It has been established that the C lobe of CaM has higher affinities but slower binding kinetics for calcium ions than the N lobe [11, 20, 23, 31]. Therefore, it has been proposed that the rate-limiting event for CaM opening is the calcium binding to its C lobe [26, 32, 67]. However, our simulation results showed the contrary. When there is no CaM-binding partner, it is the opening of N lobe requires higher calcium spike frequencies and larger free calcium elevations than the C lobe (Fig 6a). This is because the N lobe has fast calcium-binding rates and low binding affinities, resulting in rapid calcium dissociation. Hence, the calcium binding to the N lobe follows calcium spikes too close to stabilize the open conformation before the decline of free calcium concentration.

We further showed that Ng synchronizes the openings of CaM lobes at a specific calcium spike frequency. This is because Ng preferentially binds to the C lobe of CaM at low-frequency calcium spikes and releases it at high-frequency stimulation when the N lobe is opening (Fig 6b). This synchronization minimizes CaM opening during weak calcium stimulation, reduces basal CaN activity, and enhances CaMKII activation when calcium signal is strong (Figs 7 and 9).

Our findings are in agreement with studies proposing that Ng and other IQ motif proteins act as CaM caches, enhancing the activation of CaM-binding partners when the elevation of free calcium concentration is high [42, 68, 71]. Our study further shows that this increased free calcium concentration is the result of the increased calcium spike frequency, other than total calcium ions. Furthermore, the mechanisms underlying this dynamic regulation can be explained by the allosteric regulation of CaM lobes and the reciprocal influence between CaM and its binding partners.

Our frequency-response curves (Fig 9a) match with experimental observations by Huang *et al*. [16], where the absence of Ng facilitates LTD but impairs LTP induction, resulting in a down-shifted BCM curve. Upon Ng’s removal, our BCM-like curve also shifts towards low frequency calcium spikes, indicating a potentially easier onset of LTP at intermediate frequencies. This finding is in partial agreement with what has been published by Krucker *et al*. [19], a major experimental study questioning Ng’s positive role on synaptic plasticity. In their work, the mouse brains without Ng expression display lowered CaMKII activation after a high frequency train of electrical stimulation, and an easier onset of LTP under stimulation at intermediate frequencies [19]. Krucker *et al*. also showed that without Ng, LTP is enhanced at high spike frequencies, which is opposite from what was observed by Huang *et al*. and by our simulation result. Our research can not explain this discrepancy directly, because of our simplified representation of LTP. Nevertheless, we showed that in the absence of Ng, lowered CaMKII activity does not abolish LTP induction at high-frequency calcium stimulation. We further revealed that the LTP-induction frequency and the amplitude of LTP can be dynamically regulated by the expression levels of CaN and Ng (Figs 11 and 12).

The most intriguing finding from our research is that CaN mediates Ng’s regulatory role in the LTP induction at high frequency calcium spikes. CaN is one of the highly expressed protein phosphatases in the nervous system [72], and its dysfunction has been associated with many neurological diseases [73–76]. It has been shown that CaN involves in processes weakening synaptic connections [77–82]. And blockage of CaN has shown to encourage learning and memory [83, 84]. Our findings do not contradict these views. In fact, we showed that Ng’s positive impact on CaMKII activation and LTP induction is due to its suppressing of CaN activity, especially at the basal calcium concentration.

When Ng is knocked out, CaM is prone to be activated by the basal level of calcium, therefore elevating the resting level of CaN activity, which in turn decreasing CaMKII autophosphorylation. We further showed that it is CaN’s phosphatase activity, other than its high-affinity binding to CaM, mediates the function of Ng. Therefore, when CaN concentration is very low, the difference of CaN basal activity in the presence and the absence of Ng has little impact on CaMKII activity. On the other hand, when CaN concentration is high, its phosphatase activity obstructs CaMKII autophosphorylation even in the presence of Ng. This sensitivity to CaN concentration may provide a further explanation for conflicting experimental observations where Ng knock out did not always result in reduced LTP induction [15, 19]. Further experimental validations focusing on CaN’s contribution to Ng’s role in synaptic plasticity, will definitely provide new insights about the function of Ng and will broaden our understanding of synaptic plasticity.

One aspect to the regulations of Ng, which we did not include in our model, is the phosphorylation of Ng by the *γ* isoform of protein kinase C (PKC*γ*) [85]. Phosphorylated Ng losses its ability to bind CaM [12, 86, 87]. The activation of PKC*γ* is mediated by metabotropic glutamate receptor (mGluR), therefore being associated with low-frequency stimulation and the resulted slow calcium release. Although our study did not focus on this type of calcium signals, we could still speculate that under the stimulation of low-frequency calcium spikes, combined with the phosphorylation of Ng thereby releasing of CaM, CaN would be predominantly activated and contribute to the LTD induction [88].

In both repeated and continuous stimulation (Figs 8 and 7), only a small amount of CaMKII monomers retain long-lasting activities after the high-frequency calcium stimulation. Because of the large total number of CaMKII monomers, this corresponds to approximately 700 phosphorylated monomers, or nearly 60 CaMKII dodecamers. The NR2B-containing N-methyl-D-aspartate (NMDA) receptors are responsible for binding Thr286-phospho-CaMKII [89], and are present in the PSD in a small quantity [90]. These active CaMKII dodecamers are sufficient to saturate C terminal tails of NR2B containing NMDA receptors in the PSD, therefore facilitating AMPA receptor insertion and LTP induction.

This also reveals a limitation in our current approach to approximate CaMKII autophosphorylation rates, which is to estimate the probability for a given monomer to have an active neighbor. Our dendritic-spine model contains a large number of CaMKII monomers, representing a considerable amount of hexamers. Since we assumed a single homogeneous compartment, all monomers possess an equal chance to interact with CaM. Therefore, a substantial amount of active CaM molecules are required to activate two adjacent CaMKII monomers of a hexamer ring, in order to initiate trans-autophosphorylation. An improved approach would be to consider the PSD as a spatially confined compartment that is separated from the rest of the dendritic spine. In such a multi-compartment model, we could incorporate the clustering and translocation of CaMKII dodecamers, which could increase the chance of the PSD-located CaMKII to bind CaM and potentially provide a more accurate estimation of CaMKII autophosphorylation rates. However, the current low estimation of CaMKII autophosphorylation rates does not affect our major findings, which are based on the comparison of CaMKII activation in the presence and absence of Ng, and the rate of CaMKII autophosphorylation is adjusted in the same way for both situations. The only difference could be that the frequencies we found to be able to induce LTP might be higher than reality, albeit for both scenarios.

We have built our model based on a highly simplified concept, in which the effectiveness of the phosphatase and the kinase is directly used as a measure for synaptic plasticity. Although the requirements of CaN and CaMKII for the induction of synaptic plasticity are well established [9, 38], there are more modulators and interactions involved in different stages of memory consolidation [9]. The difficulties of incorporating more molecular interactions in the model are in estimating their relevant equilibrium and kinetic constants systematically and ensuring these parameters are identifiable, which we tried to ensure in this study. Our mathematical model can be expanded and parameters can be reused in future studies to further our understanding of CaM and its binding proteins.

In conclusion, our study revealed complex and dynamic roles Ng plays in regulating bidirectional synaptic plasticity. Ng functions via preferential binding to CaM C lobe at the closed conformation. And by doing so, Ng synchronizes the openings of two CaM lobes, limiting CaMKII activation at intermediate calcium spike frequencies, while facilitating it at high frequencies. Apparently contradictory experimental observations regarding its effect on LTP induction might be all valid. As we already showed before [10], the exact CaMKII activity required to induce LTP is dependent on conditions and other molecular components inside the postsynaptic dendritic spine. Ng’s positive regulation of CaMKII activity depends on both its concentration and that of CaN. Furthermore, it depends on calcium spike frequencies and the threshold CaMKII activity required to facilitate AMPA receptor insertion and LTP induction.

## Supporting information

S1 Fig Estimations of boundaries for allosteric parameter cC and calcium affinity to a binding site on CaM’s C lobe (KCT) and N lobe (KAT), as well as affinity between CaMKII peptide and open CaM (Kd_CaMKIIpeptide_RR). (EPS)

S2 Fig Estimations and correlations of allosteric parameters cC, cN, LC, LN, and affinity between Ng and closed CaM (Kd_Ng_TT). (EPS)

S3 Fig Steady state validation of calcium binding CaM. Comparison of steady state simulation results (solid lines) and published experimental observations (dots) of calcium binding C lobe (a) and N lobe (b) of CaM. To compare with experimental data published by Hoffman *et al*. [26], the model was set up with [CaM] = 5 μM and [Ng] = 50 μM. To compare with experiments published by Evans *et al*. [6], model initial states were set up as [CaM] = 2 μM and [CaMKII] = 10 μM.
(EPS)

S4 Fig Linear relationship between log10(cC) and log10(LC). (EPS)

S5 Fig Estimations of CaN binding (kon_PP2B_CaM) and releasing open CaM (koff_PP2B_CaM). (EPS)

S6 Fig Estimation of allosteric parameter cC and association constants for CaM binding CaMKII (kon_CaMKII), phospho-CaMKII (kon_CaMKIIp) and Ng (kon_Ng), calcium binding C lobe of CaM (kon_CT) and the conformational transition rate of C lobe, when no calcium or protein is bound (k_T2R_C0). (EPS)

S7 Fig Correlation analysis of each pair of the parameters estimated above in S6 Fig, based on parameter values searched and their scores for fitting experimental observations. (EPS)

S8 Fig Validations of calcium dissociation kinetics from CaM. Calcium dissociation from C-lobe of CaM (a,b) were measured upon mixing calcium chelator EGTA (10^−2^ M), and simulated after reaching equilibrium with pre-mixed CaM (10 μM) and calcium (100 μM) with (cyan) or without (salmon) Ng (50 μM). Calcium dissociation from whole CaM (c) were measured upon mixing calcium chelator quin2 (150 μM), and simulated after reaching equilibrium with pre-mixed CaM (2 μM) and calcium (20 μM) with CaMKII (2 μM salmon) or phospho-CaMKII (2 μM cyan). Solid line: simulation results; dots: experimental observations [26, 31].
(EPS)

S9 Fig Calculation of the rate of CaMKII autophosphorylation. The procedure for calculating the rate of CaMKII autophosphorylation rate as a function of active CaMKII monomers (for details see Methods section).
(EPS)

S10 Fig Protein activation after calcium inputs at 30 Hz. Computational models with (a) or without (b) Ng were stimulated by 300 calcium spikes at 30Hz. Protein activities were normalized by their total concentration, and CaMKII and CaN were further multiplied by their catalytic efficiencies towards GluR1 subunit of AMPAR. [CaM] = 40 μM, [Ng] = 40 μM, [CaN] = 8 μM, [CaMKII] = 80 μM; *kcat*_CaMKII_ = 2 s^-1^; *kcat*_CaN_ = 0.5^-1^.
(EPS)

S11 Fig Protein activation after calcium inputs at 100 Hz. Computational models were simulated with (a) or without (b) Ng, with 300 calcium spikes at 100 Hz. Each calcium input was as described in Fig 3. Both protein activities were normalized to their total concentration, then multiplied by their catalytic constant for GluR1. [CaM] = 40 μM, [Ng] = 40 μM, [CaN] = 8 μM, [CaMKII] = 80 μM; *kcat*_CaMKII_ = 2 s^-1^; *kcat*_CaN_ = 0.5^-1^.
(EPS)

S12 Fig Ng affects basal activity of CaN. Protein activity change, triggered by the train of calcium spikes (as described in Fig 7), and its basal level, were integrated over time (3000 s) and plotted as a function of calcium input frequency. Models were stimulated either with (grey circle) or without Ng (black cross). All protein concentrations were listed in Table 1.
(EPS)

S1 File Computational model. The computational model, including calcium stimulation, was encoded in Systems Biology Markup Language (SBML) Version 1 Level 3.
(XML)
